# The Local Honorary Secretary

**Published:** 1918-09-28

**Authors:** 


### THE MATRONS' AND SISTERS' DEPARTMENT.

### THE LOCAL HONORARY SECRETARY.

On the local secretary rests the responsible work of getting into touch and keeping in touch with members of the College resident in the Centre, of calling meetings of committees when requested to do so, and of keeping the minutes of the meetings which are held. She may or may not collect the subscriptions, this being a matter of arrangement with the honorary treasurer. On the untiring patience and vigilance of the honorary secretary the Centre must depend in great measure for its success. She will have plenty to do, and may need a co-adjutor or voluntary clerk.

The smooth working of any organisation governed by committees depends much on the way the minutes are kept. If the lady appointed to the post of honorary secretary has had no previous experience in minute-taking she will save herself and her colleagues much heart-burning if she take steps to get instructed in the art by someone who really understands it. Verbose records of remarks made at the meetings are no good at all and only lead to disputes. A clearly-thought-out agenda is the backbone of the minute-book, which is or should be a record of things done, not of things said. Its pages should show in exact order what motions were moved and seconded, by whom and with what result, what business was transacted, what reports received, who was chairman, and what members were present, with date and place of meeting.

In getting into touch with members of the College of Nursing within her sphere the secretary is working on almost untilled soil. She will have but small funds to depend on for postage and printing, and must look to voluntary help for the item known as "clerical assistance." How can she get to know what members of the College are at work in the Centre? Nurses live in a continual state of moving on. If they stay in one neighbourhood two or three years it is the exception. Half the names on her list of members may be changed before the year is out, and twice as many more new members of the College may have come meantime to live in the Centre. Yet it is a chance if one in ten will acquaint the long-suffering secretary with her change of address. These facts must be recognised from the outset or there will be bitter disappointment. Each Centre must lay itself out to draw the ever-flowing stream of nurses into the channel formed through the beneficent action of the College. All members of the College must be drawn in and, if possible, helped, and encouraged professionally as they pass through, but very few indeed can be depended on for continuous service.

There is one helpful feature as regards getting into touch with members. It is that nurses seldom while in active work lack a business address. Private nurses, for instance, are sent out from an institute or live for the most part when not at their cases at private hotels or boarding houses adapted to their day and night requirements. Accordingly the secretary must take some trouble to compile a list of the hostels frequented by nurses, of the nursing institutes within their boundaries, of the hospitals, Poor-Law infirmaries, municipal institutions, district nursing centres and the like. To each of these places there should be sent a framed card giving the official address of the Centre, and requesting members of the College of Nursing to communicate with the secretary, with a view to receiving notices of meetings, etc. If voluntary help be forthcoming to hand-print these cards in an attractive form there will be few refusals to give them a place where all nurses may see them. In a great many institutions and homes some member will be found willing to act as correspondent and distribute notices, send in new names, and advise the secretary of changes in the list of her members. Thus with the least possible expenditure of stamps and writing nurses who pass through the district will come to hear about the Centre and have an opportunity of joining it. The value of securing a correspondent in every spot where nurses are to be found will be proved whenever it is desired to hold meetings not merely of members of the College but of nurses in general. Every zealous correspondent is an important thread in the fabric of unity of which the College is to be woven.

It is the particular privilege of the Local Centres to minister to the ever-moving nursing population. Hard as they may seem, the duties of the Centres towards those ships who pass in the night must never be foregone. The weary complaint, "What is the good of it? We shall never see her again," must never be heard. What if she passes on and vanishes? Perhaps the welcome given her at the College Centre remains with her as the one bright spot through a troubled time. Is not that enough?

